# Passive heat therapy is feasible but does not affect cardiometabolic health outcomes in persons with spinal cord injury – a pilot study

**DOI:** 10.1113/EP093636

**Published:** 2026-04-25

**Authors:** Sven P. Hoekstra, Christof A. Leicht, Dean Kellogg, Yubo Wu, Terry Romo, Hannah Uhlig‐Reche, Michelle Trbovich

**Affiliations:** ^1^ Department of Rehabilitation Medicine University of Texas Health at San Antonio San Antonio Texas USA; ^2^ Department of Exercise and Sport Science St. Mary's University San Antonio Texas USA; ^3^ The Peter Harrison Centre for Disability Sport, School of Sport, Exercise and Health Sciences Loughborough University Loughborough UK; ^4^ South Texas Veteran's Health Care System San Antonio Texas USA; ^5^ Department of Medicine University of Texas Health at San Antonio San Antonio Texas USA

**Keywords:** cardiometabolic health, glucose tolerance, inflammation, microvascular function, passive heating, thermal interventions

## Abstract

Passive heat therapy can improve cardiometabolic health outcomes in some clinical populations, making it a potential therapeutic tool for individuals with spinal cord injury (SCI), who exhibit elevated cardiometabolic disease risk and face barriers to physical activity. Following a no‐intervention run‐in of 8 weeks (Control), 10 adults with chronic SCI (age: 48 ± 14 years; lesion level: C4–T12, body mass index: 29 ± 2) attended thrice weekly sessions over 8 weeks (Intervention), during which sublingual temperature was raised by 1°C using a water‐perfused suit and heated blankets. An oral glucose tolerance test, resting plasma interleukin 6, C‐reactive protein and tumour necrosis factor α concentration, as well as the skin hyperaemic response to local heating of 39°C and 45°C were assessed at baseline, after Control and Intervention. Adherence, safety and tolerability was monitored throughout the intervention. While one participant dropped‐out after 18 intervention sessions due to a medical issue unrelated to the intervention, adherence was 100% in the remaining nine participants and tolerated well. Glucose tolerance did not differ between Control and Intervention (glucose area under the curve Control: 7410 ± 6088 mg/dl*180min, Intervention: 8253 ± 2139 mg/dl*180min; *P* = 0.857). Similarly, neither inflammatory markers (*P* > 0.055) nor hyperaemic responses to local skin heating (*P* > 0.829) changed after intervention. In summary, passive heat therapy that raised sublingual temperature by 1°C was well tolerated, but did not change outcomes associated with cardiometabolic health and inflammation in persons with chronic SCI.

## Abstract (highlights)

- **What is the central question of this study?**
  Is passive heat therapy, consisting of elevating sublingual temperature by 1°C using a water‐perfused suit and heating blankets, feasible and can it improve cardiometabolic health outcomes in persons with spinal cord injury (SCI)?
- **What is the main finding and its importance?**
  The main finding of this study is that passive heat therapy is well tolerated by persons with SCI. However, we found limited improvements in inflammation, microvascular function and glucose tolerance, indicating that modifications to the heating protocol may be warranted.

## 1 INTRODUCTION

Acute heat exposure, experienced during sauna sessions or hot baths, can trigger physiological responses similar to those observed during exercise. For instance, exercise and heat exposure both induce sweating, increase heart rate, shear stress and cardiac output (Brunt et al., 2021), and both modalities can elevate adrenaline and interleukin (IL)‐6 concentrations (Laing et al., 2008). Such transient perturbations may underpin chronic adaptations. However, whilst these acute responses are relatively well described, there remains a need for chronic intervention studies, both in healthy and in clinical populations. Nonetheless, the available evidence indicates that, much like regular exercise, heat therapy has the potential to enhance aspects of cardiometabolic health (Brunt et al., 2021, Price et al., 2025), which is particularly relevant for populations with physical challenges to participation in recommended levels of physical activity.

Repeated hot water immersion (HWI) has been shown to reduce fasting blood glucose in individuals with type 2 diabetes (T2D) (Hooper 1999) and overweight men (Hoekstra et al., 2018), reduce glycated haemoglobin (HbA1c) in patients with hypertension and obesity (Olah et al., 2011), and improve glucose and insulin area under the curve (AUC) following an oral glucose tolerance test (OGTT) in obese women with polycystic ovary syndrome (Ely et al., 2019). Further, improved endothelial and cardiac function in chronic heart failure (Kihara et al., 2002), lower resting plasma IL‐6 concentrations in chronic heart failure (Oyama et al., 2013), and lower systolic and diastolic blood pressure (BP) across several investigations (Price et al., 2025) are reported following heat therapy. Epidemiological data further show a negative relationship between frequency of sauna bathing and systemic inflammation (Laukkanen et al., 2018); in addition, the frequency of habitual hot bathing is associated with reduced BP and HbA1c in patients with T2D (Katsuyama et al., 2022), and with a lower cardiovascular disease risk (CVD) in the general population (Ukai et al., 2020). However, whilst these findings provide support for the therapeutic potential of heat therapy, they must also be seen in context of studies failing to document heat therapy‐induced changes to vascular function (Price et al., 2025) or markers associated with inflammation (Hoekstra et al., 2018, Olah et al., 2011).

A spinal cord injury (SCI) is associated with active muscle loss and reduced levels of physical activity, predisposing individuals with SCI to chronic low‐grade inflammation, a CVD risk factor characterised by elevated resting levels of pro‐inflammatory cytokines (Davies et al., 2007). Individuals with SCI further exhibit impaired glucose tolerance (Yarar‐Fisher et al., 2013) and have a 2.3 times higher risk for T2D compared with the non‐SCI population (Tavakoli et al., 2025). This may be driven by a combination of factors specific to SCI, including reduced active skeletal muscle mass, higher levels of adiposity, impaired sympathetic nervous system activity, and limited capacity or access to engage in exercise of sufficient intensity or duration (Farkas et al., 2022). In addition, individuals with SCI exhibit reduced arterial diameter and endothelial function compared with the non‐SCI population (Thordarson et al., 2025).

Thermoregulation, and potentially some of the physiological responses to heat exposure, may differ in individuals with SCI, given their reduced muscle mass, alterations in local blood flow, as well as sympathetic dysfunction, impacting on the sweat and adrenaline response (Price and Trbovich, 2018). Nonetheless, favourable changes in shear patterns (Coombs et al., 2019), and acute elevations in IL‐6 and IL‐1 receptor antagonist (IL‐1ra) despite an absent adrenaline response after a bout of acute exposure have been reported in SCI (Hashizaki et al., 2018; Leicht et al., 2015). Although these acute findings are encouraging, no study to date has examined the chronic effects of repeated heat exposure in the SCI population. Whether an extended heat intervention can reduce markers associated with inflammation, improve glycaemic control, or be tolerated safely in this group remains unknown. Given the elevated CVD risk in SCI, the physiological rationale linking heat exposure to inflammation‐related and metabolic improvements, and the challenges around exercise in this group, heat therapy represents a compelling potential strategy.

Therefore, this pilot study investigates the effects of an 8‐week chronic heat therapy intervention in individuals with SCI. We aimed to examine (1) safety, tolerability and perceptual responses, (2) changes in resting inflammatory markers and endothelial function, and (3) alterations in glycaemic control in response to an OGTT to repeated heat exposure. We hypothesize that heat therapy reduces resting pro‐inflammatory cytokine concentrations, improves glucose tolerance, and is well tolerated in individuals with SCI.

## 2 METHODS

### 2.1 Participants

A convenience sample of adult Veterans with chronic SCI was recruited from the outpatient clinic of the Audie L. Murphy Memorial Veterans Hospital in San Antonio. Potential participants were identified by physician–researcher M.T. using the hospital clinical database. We excluded participants who were current smokers, those on daily anti‐inflammatory or vasoactive medications, those currently suffered from pressure ulcers or skin breakdown, those with a history of heat‐related illness, any active acute illness, or a baseline haemoglobin concentration of less than 11 g/dl documented within 6 months of study initiation. After having been provided information about the study procedures, approved by the University of Texas Health Science Center at San Antonio Institutional Review Board (Study 20220185H) in line with the 2013 version of the *Declaration of Helsinki*, and registered at clinicaltrials.gov under ID NCT04971408, participants provided written informed consent.

### 2.2 Study design

**FIGURE 1:**
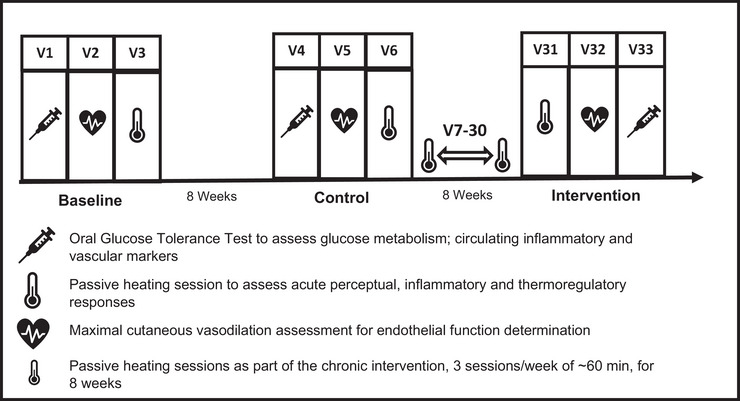
Schematic representation of the study design.

In this pilot study, participants engaged in two study phases, by which they served as their own control. Outcome measures were assessed at the first three laboratory visits (Baseline), after 8 weeks without intervention (Control) and 2–4 days following an 8‐week passive heat therapy (PHT) intervention (Intervention; Figure 1). The intervention consisted of thrice weekly PHT, during which sublingual temperature was elevated by 1°C. All procedures took place in the Audie L. Murphy Memorial Veterans Hospital in San Antonio. The study was part of the same project as described previously (Uhlig‐Reche et al., 2025). Here, the primary outcome was feasibility, with inflammatory, vascular and glycaemic measures as exploratory outcomes.

### 2.3 Procedures

#### 2.3.1 Visit 1: OGTT and inflammatory marker concentration

Participants visited the Clinical Research Unit at the Veteran Affairs Hospital in a fasted state for a 3‐h OGTT. Medication was allowed prior to the visit. First, a peripheral intravenous catheter was placed for recurrent blood draws. Following two baseline blood draws, participants consumed a 75 g glucose drink within 5 min. Thereafter, blood samples for the determination of glucose, insulin and c‐peptide concentration were collected at 15, 30, 45, 60, 90, 120, 150 and 180 min post‐drink, according to Trbovich et al. (2025). The incremental AUC was calculated using the trapezoid method. Throughout the procedures, participants rested in a semi‐supine position. Two participants remained in their wheelchair during the assessment. The first fasting blood draw was also used for the determination of plasma IL‐6, tumour necrosis factor α (TNF‐α), C‐reactive protein (CRP) and IL‐1ra concentration. Plasma CRP, IL‐6, TNF‐α, glucose, insulin and c‐peptide concentrations were analysed in the central laboratory of the Audie L. Murphy Memorial Veterans Hospital immediately upon sample collection by enzyme‐linked immunoassays (ELISA; Roche Cobas E801). For the IL‐ra analysis, whole blood was centrifuged for 10 min at 4°C and 1500 *g*. IL‐1ra concentration was then assessed in batch, using a commercially available enzyme‐linked immunoassay (R&D Systems, Minneapolis, MN, USA). The intra‐plate coefficient of variation was 4.4%. Intracellular heat shock protein 72 expression measured in monocytes was pre‐registered as an outcome measure, but we did not obtain viable data due to technical difficulties with the flow cytometry protocol.

#### 2.3.2 Visit 2: Local thermal hyperaemia

Local thermal hyperaemia was measured as an assessment of endothelial function in the microvasculature. Endothelial function was assessed via cutaneous vasodilatory responses in response to local skin heating at 39°C and 45°C. Laser Doppler flowmetry (LDF) was used to measure skin blood flow using the following equation: cutaneous vascular conductance (CVC) = LDF/mean arterial BP. Specifically, with participants resting in a supine position, LDF probes were placed on the lower leg and arm. Following a 10 min baseline phase at a probe temperature of 34°C, both probes were heated to 39°C at a rate of 0.1°C/s, kept at that temperature for 30 min, then further increased to 45°C at 0.1°C/s, and maintained for 30 min (Choi et al., 2014). Beat‐by‐beat BP was obtained at the fingertip throughout the procedure (Finapres Medical Systems, Enschede, Netherlands). The average CVC of the final 3 min of each temperature phase, sampled at 10‐s intervals, was used for analyses. Data were measured at 34°C (baseline), and 39°C (NO‐dependent vasodilation), then 45°C (maximum vasodilation). The CVC at 39°C was normalized as a percentage of maximal vasodilation (i.e., the absolute CVC at 45°C), while the CVC at 45°C as an outcome measure was normalized as a percentage of baseline CVC (Choi et al., 2014).

#### 2.3.3 Visit 3: Acute responses to PHT

Employing a passive heating session identical to those in the intervention phase (see ‘Intervention’), the acute thermo‐physiological, perceptual and inflammatory response to passive heating was assessed. Following 15 min of supine rest, baseline measures were obtained, and a resting blood sample was collected by venipuncture of an antecubital vein. Blood samples were processed and analysed as described under Visit 1.

Physiological and perceptual outcomes were then assessed during heating every 15 min. Deep tissue temperature at the vastus lateralis was measured using the validated non‐zero heat flux method (Bair Hugger, 3M, Minnesota, MN, US). Skin temperature (*T*_skin_) was measured by local thermocouples over the chest, upper arm, and upper and lower leg. BP and pulse rate (heart rate, HR) were measured via Portapres (Finapres), while sublingual temperature was monitored by a probe placed under the tongue for 5 min prior to each measurement. Thermal sensation was measured on a 9‐point Likert scale, thermal comfort on a 5‐point Likert scale (Gagge et al., 1967), and basic affect using the Feeling Scale (Hardy & Rejeski, 1988). Once a rise in sublingual temperature of 1°C was achieved, the session was terminated, and a second blood sample was obtained immediately after.

Visit 4, 5 and 6, completed following 8 weeks of no intervention (Control), were identical to visits 1, 2 and 3, except for the lack of blood sample collection during visit 6.

### 2.4 Intervention

During Intervention, participants visited the laboratory thrice weekly (visits 7–31). Prior to each session, baseline sublingual temperature was obtained by sublingual placement of a temperature probe for 5 min. Thereafter, with participants in a supine position, a water‐perfused suit with the infused water set at 48°C was placed over the torso, while three heated fleece‐lined electrical blankets set at 43°C and an aluminium foil blanket were placed over the entire body. Each session was completed when sublingual temperature was elevated by 1°C from baseline, or when the participant requested termination due to thermal discomfort. At completion of each session, participants rated their thermal sensation, thermal comfort and affect, and were provided with 250 mL of water whilst they remained in the laboratory for 15 min for clinical monitoring.

The procedures of visits 31, 32 and 33 were identical to those of visits 1, 2 and 3, except for the lack of blood sample collection during the acute responses to the PHT visit. However, the order was different in that the acute responses to PHT were assessed in the final session of the Intervention phase (visit 31), followed by the two visits to conduct the OGTT (visit 32) and local thermal hyperaemia assessment (visit 33).

### 2.5 Intervention feasibility

To facilitate adherence, the time of day for visits 4–30 was allowed to vary within participants, and participants did not receive dietary instructions prior to their visits. If required, transportation to the laboratory was arranged. All visits were recorded in participants’ clinical records, including any potential adverse events and serious adverse events. The occurrence of autonomic dysreflexia (AD) (systolic BP over 150 mmHg or an increase of at least 20 mmHg from baseline), symptomatic orthostatic hypotension (OH) (self‐reported light‐headedness or dizziness when changing from a supine to sitting position associated with a drop in systolic BP of 20 mmHg) and skin breakdown due to burns from the heating blankets/suit were monitored specifically. Study fidelity was assessed by monitoring adherence and tolerability. Session adherence was satisfied if participants visited the laboratory for their session, whilst a session was considered tolerable if participants reached the prescribed 1°C rise in sublingual temperature.

### 2.6 Statistical analysis

Statistical analyses were conducted using the 28th version of SPSS Statistics (IBM Corp., Armonk, NY, USA). Data are presented as means ± SD unless otherwise stated. Normality was checked by a combination of the Shapiro–Wilk test and visual inspection of the raw data. The acute changes in outcomes following the PHT session at Baseline were assessed using Student's paired *t*‐test. The effect of the chronic intervention on the acute response to a single PHT session was then assessed by comparing these responses with those at the final session of Intervention, using a paired *t*‐test. The Baseline time point (instead of Control) was chosen as participants were entirely naïve to the PHT protocol at this point in the study. The change in the outcome measures following the chronic intervention was analysed using a repeated measures ANOVA comparing the three assessment time points (Baseline, Control, Intervention), with a Bonferroni‐corrected *post hoc* paired *t*‐test as follow‐up. The inflammatory marker and thermal hyperaemia analyses were performed on all 10 participants, the other outcomes on nine participants. Statistical significance was accepted at *P* ≤ 0.05.

## 3 RESULTS

### 3.1 Participants

After screening 13 potential participants, 10 adults with chronic SCI were enrolled in the study between January 2023 through July 2024 (Table 1). They were predominantly male (*n* = 8), with an ASIA score from A through D, 48 ± 14 years old and 12 ± 12 years following their spinal injury.

**TABLE 1:** Participant characteristics.

Parameter	Mean ± SD or range
Age (years)	48 ± 14
Sex (m/f)	8/2
Body mass index (kg/m^2^)	29 ± 2
Spinal lesion level	C4–T12
ASIA score (*n*)	
A	4
B	1
C	4
D	1
Time since injury (years)	12 ± 12

### 3.2 Acute responses to passive heating

During the first passive heating session, it took 60 ± 14 min to attain a 1°C rise in sublingual temperature. This was accompanied by an increase in HR to 89 ± 20 bpm, and a vastus lateralis tissue temperature of 36.0 ± 2.2°C, while systolic BP was reduced from 114 ± 21 to 102 ± 18 mmHg (Table 2). Participants reported an 8 ± 1 on the thermal sensation scale, and a 2 ± 3 on the Feeling Scale by the end of the session. None of the inflammatory markers were elevated immediately following the session (Table 2; *P *> 0.182).

**TABLE 2:** Acute physiological, perceptual and inflammatory response to a single passive heat therapy session at baseline and the final session of intervention.

Parameter	Baseline pre	Baseline end	*P* time	Intervention pre	Intervention end	*P* time	*P* session
Time to Δ1°C (min)	N/A	60 ± 14	N/A	N/A	63 ± 17	N/A	
Mean skin temperature (°C)	32.9 ± 1.0	38.1 ± 1.2	**< 0.001**	32.8 ± 1.0	38.1 ± 0.7	**< 0.001**	0.670
Calf skin temperature (°C)	30.9 ± 1.7	38.1 ± 2.1	**< 0.001**	30.8 ± 1.5	37.7 ± 1.1	**< 0.001**	0.987
Thigh skin temperature (°C)	32.7 ± 2.5	37.5 ± 1.8	**< 0.001**	31.6 ± 2.0	37.1 ± 1.1	**< 0.001**	0.908
Chest skin temperature (°C)	34.1 ± 1.1	38.3 ± 0.8	**< 0.001**	34.3 ± 1.2	38.6 ± 0.7	**< 0.001**	0.445
Arm skin temperature (°C)	33.1 ± 1.6	38.4 ± 1.0	**< 0.001**	33.5 ± 1.4	38.6 ± 0.5	**< 0.001**	0.660
Vastus lateralis tissue temperature (°C)	33.3 ± 1.8	36.0 ± 2.2	**0.001**	32.5 ± 1.5	35.7 ± 1.6	**0.002**	0.259
Systolic blood pressure (mmHg)	114 ± 21	102 ± 18	**0.049**	123 ± 20	116 ± 21	0.395	0.113
Diastolic blood pressure (mmHg)	66 ± 12	63 ± 14	0.426	72 ± 14	68 ± 13	0.361	0.469
Pulse rate (bpm)	73 ± 12	89 ± 20	**0.004**	74 ± 14	87 ± 16	0.003	0.397
Thermal sensation (1 to 9)	5 ± 1	8 ± 1	**< 0.001**	5 ± 1	6 ± 1	0.117	**0.007**
Thermal comfort (0 to 5)	0 ± 0	2 ± 2	**0.013**	0 ± 0	0 ± 1	0.200	0.069
Basic affect (–5 to +5)	2 ± 2	2 ± 3	0.882	4 ± 2	4 ± 2	0.356	0.162
Interleukin‐6 (pg/ml)	7.96 ± 5.34	7.50 ± 6.46	0.516	Not measured	Not measured	N/A	N/A
C‐reactive protein (ng/ml)	0.66 ± 0.55	0.70 ± 0.63	0.418	Not measured	Not measured	N/A	N/A
Tumour necrosis factor‐α (pg/ml)	1.05 ± 0.46	1.00 ± 0.38	0.276	Not measured	Not measured	N/A	N/A
Interleukin‐1 receptor antagonist (pg/ml)	608 ± 199	798 ± 298	0.182	Not measured	Not measured	N/A	N/A

The Time *P*‐values are obtained through a paired *t*‐test between Pre and End, while the Session *P*‐value is obtained through a paired *t*‐test on the End values at Baseline and Intervention. Statistically significant differences are highlighted in bold.

The acute physiological responses during the final session of Intervention were similar compared to those at Baseline (Table 1). The 1°C rise in sublingual temperature was achieved after 63 ± 17 min (*P* = 0.247). However, the thermal perceptual responses at the end of the final session of Intervention tended to be more positive compared to the ratings at the Baseline session (TS: 8 ± 1 vs 6 ± 1, *P* = 0.007; TC: 2 ± 1 vs. 0 ± 1, *P* = 0.069).

### 3.3 Feasibility of the chronic intervention

All participants completed the control phase. One participant dropped out after 18 passive heating sessions of the intervention phase for reasons unrelated to the study. The remaining nine participants completed all their prescribed intervention sessions. Out of the 234 sessions completed overall, 12 sessions were terminated after a sublingual temperature rise of 0.8°C due to thermal discomfort. Two participants, who used an electronically powered wheelchair, completed the passive heating sessions whilst remaining in a reclined position in their wheelchair due to personal preference and comfort. The other eight participants completed the sessions as intended, in a supine position on a hospital bed.

During the intervention phase, three participants reported signs of mild constipation that were easily treated. There were no other study‐related adverse events; this included no signs of AD, OH or skin damage from burns.

### 3.4 Preliminary efficacy of the chronic intervention

**FIGURE 2:**
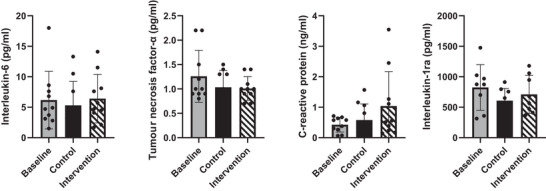
Resting inflammatory marker concentrations at Baseline, Control and Intervention. Black circles are individual data points; the bar and error bar signify the mean and standard deviation. The *P*‐values displayed were obtained through one‐way repeated measures ANOVA.

Resting systolic BP (Baseline: 114 ± 21 mmHg, Control: 117 ± 19 mmHg, Intervention: 123 ± 20 mmHg; *P* = 0.521), HR (Baseline: 72 ± 10 bpm, Control: 76 ± 12 bpm, Intervention: 74 ± 14 bpm; *P* = 0.633) and sublingual temperature (36.6 ± 0.3°C, 36.6 ± 0.2°C, 36.6 ± 0.4°C; *P* = 0.879) were unchanged following Intervention compared with Baseline and Control. Furthermore, compared with Baseline and Control, Intervention did not change fasting plasma IL‐6, TNF‐α, CRP or IL‐1ra concentrations (Figure 2; *P *> 0.055).

**FIGURE 3:**
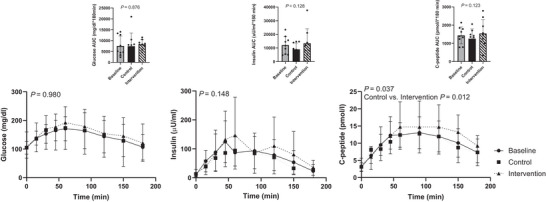
Glucose, insulin and c‐peptide concentrations during the oral glucose tolerance test at Baseline, Control and Intervention. Inserts are the incremental area of the curve for each respective analyte. Black circles are individual data points; the bar and error bar signify the mean and standard deviation. The *P*‐values displayed on the AUC graphs were obtained through one‐way repeated measures ANOVA. The *P*‐values displayed on the time course data were obtained through two‐way (condition × time) repeated measures ANOVA for all three oral glucose tolerance tests, unless stated otherwise.

Fasting glucose (*P* = 0.836) and insulin (*P* = 0.328) concentration were not altered after Intervention compared with Baseline and Control. Similarly, the time course of the glucose (*P* = 0.980) and insulin (*P* = 0.148) concentrations during the OGTTs was not different between Baseline, Control and Intervention (Figure 3). However, plasma c‐peptide concentrations during the OGTT were higher after Intervention compared with Control (*P *= 0.012), but not Baseline (*P *= 0.171). The glucose (*P* = 0.876), insulin (*P* = 0.128) and c‐peptide (*P* = 0.123) AUCs were unaltered after Intervention (Figure 3).

**FIGURE 4:**
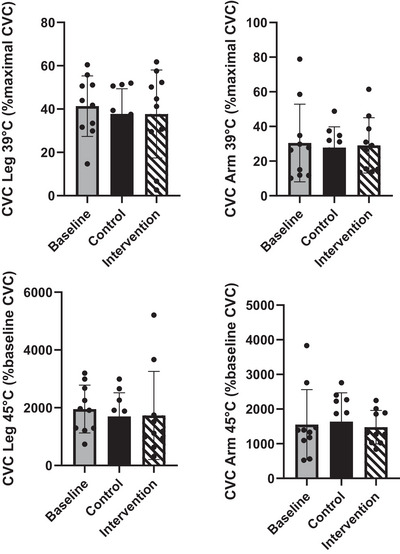
Cutaneous vascular conductance at the arm and leg in response to local heating at 39°C and 45°C at Baseline, Control and Intervention. Black circles are individual data points; the bar and error bar signify the mean and standard deviation. The *P*‐values displayed were obtained through one‐way repeated measures ANOVA.

The local thermal hyperaemic response to 39°C (*P* = 0.838) and 45°C (*P* = 0.829) heating at the level of the leg was not different between Baseline, Control and Intervention (Figure 4). The same was true for the assessments conducted at the arm (39°C, *P* = 0.876; 45°C, *P* = 0.875).

## 4 DISCUSSION

Persons with SCI live with an increased risk for cardiometabolic disease (Cragg et al., 2013) paired with numerous physical and environmental barriers to engage in regular exercise (Williams et al., 2014). At the same time, SCI is known for autonomic dysfunction‐related thermoregulatory impairments. Thus, while an accessible intervention such as PHT may prove clinically beneficial in this population, conservative implementation of heating protocols is warranted. Employing a relatively modest heat stimulus, this chronic pilot trial in persons with SCI indicates that PHT is feasible and safe to use in this population. However, the lack of meaningful change in any of the preliminary efficacy outcomes suggests that alterations to the heating protocol may be necessary when embarking on a larger follow‐up trial.

### 4.1 Acute responses to passive heating

As PHT is yet relatively unexplored in persons with SCI, we first characterized the acute physiological and inflammatory responses to a single heat exposure with a sublingual temperature rise of 1°C. In contrast to the lack of acute change reported by a recent meta‐analysis investigating predominantly able‐bodied individuals (Price et al., 2025) and other studies in SCI (Coombs et al., 2019; Hashizaki et al., 2018; Leicht et al., 2015), systolic BP was reduced by 12 mmHg at the end of the session. The reduced sympathetic vascular tone found after SCI (Trbovich et al., 2022) may have led to both greater vasodilation in response to heating and an impaired responsiveness of the heart to match cardiac output with the haemodynamic demands during heating. Although heart rate was increased in the current study, studies directly comparing persons with SCI and able‐bodied individuals indeed show an attenuated increase in heart rate during heating after SCI (Hashizaki et al., 2018). Surprisingly though, a similar study using a water‐perfused suit found no reduction in systolic BP in persons with complete cervical SCI (Hashizaki et al., 2018), the lesion type most affected by autonomic dysfunction. One notable difference between both studies is the substantially higher baseline BP found in the current study, likely related to the inclusion of individuals with an incomplete spinal lesion (ASIA scores C and D). Of note, there were no signs of dizziness or light‐headedness during or after the session.

In line with aerobic exercise (Petersen, 2005), part of the beneficial adaptations following PHT may be attributed to the anti‐inflammatory milieu that is created with each repeated session. However, the heating protocol used in the current study did not lead to an acute change in the inflammatory profile. There appears to be a dose–response relationship between the rise in core temperature and the acute inflammatory response, with most studies that elevate core temperature with ∼1.5°C or more consistently showing an increase in, for example, IL‐6 (Hoekstra et al., 2018, 2021, 2023; Laing et al., 2008; Leicht et al., 2015). On the other hand, a 1°C rise in core temperature has previously been shown sufficient to elevate IL‐6 concentration in AB individuals (Faulkner et al., 2017) and those with SCI (Hashizaki et al., 2018). Whilst the lack of an acute IL‐6 response to the current protocol may be related to the modest heat load, the divergent IL‐6 findings may also be related to the analysis method. Baseline IL‐6 concentrations were substantially higher in the current compared with other SCI studies (∼6 pg/ml vs. 1–2 pg/ml; Leicht et al., 2015; Hashizaki et al., 2018), with ELISAs conducted at a clinical laboratory versus the R&D Systems kit used in both other studies.

### 4.2 Feasibility of the chronic intervention

Aside from one participant that dropped out after 18 intervention session due to a medical issue unrelated to the study, adherence to the intervention was 100%, and 95% of the sessions were completed as prescribed. Additionally, there were no incidences of AD, OH or adverse events related to thermoregulatory impairments noted during the intervention. This is an important finding as the increased risk for heat illness due to an impaired thermoregulatory capacity is an often‐cited concern when exposing persons with SCI to high ambient temperatures (Trbovich et al., 2020; Griggs et al., 2019). The excellent adherence and tolerability of the intervention is likely related to the relatively mild heat exposure, as many other PHT studies raise core temperature by ∼1.5°C instead of the 1°C in the current study (Brunt et al., 2016; Hoekstra et al., 2018; Kaiser et al., 2025). Moreover, it is encouraging that the perceptual responses during heating were more positive when assessed at the end of the PHT intervention compared with the baseline assessment, suggesting that habituation took place over the 8‐week period.

Notwithstanding the promising feasibility findings, two minor adverse events that occurred during the study period are worth noting from the perspective of PHT research in persons with SCI. Firstly, three participants developed mild constipation between the second and third week of the PHT intervention. Whilst this was easily treated and did not interfere with study adherence, it may be a potential side‐effect of PHT that is worth considering in future studies. In AB individuals, constipation following PHT may be related to the heat‐induced dehydration (Arnaud, 2003). However, when measuring sweat loss in a sub‐group of participants, including those with constipation, we found only a negligible sweat loss (< 50 mL). This is expected in persons with SCI, in particular those with a high lesion level (Trbovich et al., 2021). Dehydration as such is therefore unlikely to explain the mild constipation observed. However, a redistribution of blood away from the gastrointestinal organs toward the heated skin may be a more likely potential explanation (Crandall & Gonzalez‐Alonso, 2010).

Second, this pilot study revealed a methodological consideration that is relevant for research in SCI more generally. In alignment with other SCI studies measuring core temperature during heat stress (Griggs et al., 2015), our protocol included CorTemp (HQ Inc.) gastrointestinal temperature sensors to monitor core temperature. However, the first enrolled participant, who incidentally had a colostomy, monitored stool output closely and noted that he had not passed the sensor 2 weeks following its ingestion at Visit 3. An X‐ray was ordered, which confirmed the pill was retained in the right lower abdominal quadrant. After days of bowel cleanout without success, the sensor required retrieval by colonoscopy. This incident is likely related to the neurogenic bowel and the associated longer bowel transit time common after SCI (Tate et al., 2016), and underscores that gastrointestinal temperature sensors should be used with caution in this population. As a result, we discontinued the use of the ingestible temperature sensors and utilized oral temperature monitoring during the remainder of the study.

### 4.3 Preliminary efficacy of the chronic intervention

The 8‐week PHT intervention did not meaningfully improve glycaemic control, microvascular endothelial function, or resting inflammatory profile. As described above, here we employed a relatively modest heat exposure (to ensure safety), which most likely explains these findings. A recent meta‐analysis showed sustained reductions in BP following chronic PHT interventions in AB individuals (Price et al., 2025), and studies that increased core temperature by at least ∼1.5°C found improvements in glycaemic control (Ely et al., 2019; Hoekstra et al., 2018), microvascular endothelial function (Brunt et al., 2016) and the inflammatory profile (Ely et al., 2019) following interventions of similar (Brunt et al., 2016; Ely et al., 2019) or shorter duration (Hoekstra et al., 2018). On the other hand, Kaiser et al. (2025) reported similar findings to the current study, with an acute reduction in BP following a single PHT session, but no change in resting BP, arterial stiffness or inflammatory marker concentration following an 8‐ to 10‐week PHT intervention in persons with untreated hypertension.

At the same time, there exists evidence of beneficial adaptations after PHT interventions investigating similar or more modest elevations in core temperature (Hesketh et al., 2019; Monroe et al., 2022; Ro et al., 2025). Notably, Hesketh et al. (2019) found improvements in glucose tolerance following 6 weeks of thrice weekly PHT using a heat chamber set at 40°C, during which core temperature remained stable. It is likely that the variability between persons with SCI, resulting from different lesion levels and degrees of lesion completeness as well as the associated comorbidities, reduces the statistical power to detect changes in a given outcome measure. While recruitment challenges exist in this population, follow‐up trials should consider this in their study design. At the same time, it should be noted that the current intervention did have beneficial effects in the form of a reduced perceived pain intensity as self‐reported on the SCI pain questionnaire following the intervention, which has previously been published elsewhere (Uhlig‐Reche et al., 2025). Nonetheless, considering the available evidence from AB individuals of a dose–response relationship between core temperature elevation and acute physiological responses as well as the more consistent cardiovascular improvements shown after heating protocols that elevate core temperature to a larger extent (e.g., Brunt et al., 2016; Ely et al., 2019; Price et al., 2025), the current study findings indicate that more intense or prolonged heat exposure may be needed to induce meaningful changes in cardiometabolic health outcomes. Such follow‐up studies should also consider employing a randomized controlled trail‐design, as the run‐in phase prior to the intervention in the current study is a limitation that should be kept in mind when interpreting the findings.

In conclusion, this pilot study demonstrated that laboratory‐based PHT that elevates sublingual temperature by 1°C is safe and feasible in persons with chronic SCI. The absence of meaningful changes in the assessed cardiometabolic health outcomes is likely related to the limited prescribed rise in core temperature and the small sample of individuals with various lesion levels studied. While these findings pave the way to explore PHT as a therapeutic tool in persons with SCI, follow‐up trials should consider modifications to the heating protocol and/or session frequency and intervention duration.

## AUTHOR CONTRIBUTIONS

Sven P. Hoekstra: Study Design, Data Acquisition, Data Analysis, Interpretation, Writing of Manuscript, Approval of Manuscript. Christof A. Leicht: Study Design, Interpretation, Writing of Manuscript, Approval of Manuscript. Dean Kellogg Jr.: Study Design, Data Acquisition, Data Analysis, Interpretation, Feedback on Manuscript, Approval of Manuscript. Yubo Wu: Study Design, Data Acquisition, Data Processing, Approval of Manuscript. Terry Romo: Data Acquisition, Data Processing, Participant Safety Monitoring, Approval of Manuscript. Hannah Uhlig‐Reche: Data Acquisition, Data Processing, Data Analysis, Feedback on Manuscript, Approval of Manuscript. Michelle Trbovich: Study Design, Data Acquisition, Data Analysis, Interpretation, Writing of Manuscript, Participant Safety Monitoring, Approval of Manuscript. All authors have read and approved the final version of this manuscript and agree to be accountable for all aspects of the work in ensuring that questions related to the accuracy or integrity of any part of the work are appropriately investigated and resolved. All persons designated as authors qualify for authorship, and all those who qualify for authorship are listed.

## CONFLICT OF INTEREST

None declared.

## Data Availability

Data is available upon request from the corresponding author.
